# In silico and in vitro analyses of a novel FoxO1 agonist reducing Aβ levels via downregulation of BACE1

**DOI:** 10.1111/cns.14140

**Published:** 2023-03-09

**Authors:** Ming‐Ti Lv, He‐Cheng Wang, Xiao‐Wen Meng, Ya‐Ting Shi, Yi‐Min Zhang, Lin‐Lin Shan, Ru‐Ling Shi, Tian‐Jun Ni, Ying‐Chao Duan, Zhi‐Jun Yang, Wei Zhang

**Affiliations:** ^1^ School of Basic Medical Sciences Xinxiang Medical University Xinxiang China; ^2^ School of Life and Pharmaceutical Sciences Dalian University of Technology Panjin China; ^3^ School of Medical Technology Xinxiang Medical University Xinxiang China; ^4^ School of Pharmacy Xinxiang Medical University Xinxiang China; ^5^ School of Life Science and Technology Xinxiang Medical University Xinxiang China

**Keywords:** Alzheimer's disease, BACE1, FoxO1 agonist, in silico screening, molecular dynamics simulation, β‐Amyloid

## Abstract

**Aims:**

FoxO1 is an important target in the treatment of Alzheimer's disease (AD). However, FoxO1‐specific agonists and their effects on AD have not yet been reported. This study aimed to identify small molecules that upregulate the activity of FoxO1 to attenuate the symptoms of AD.

**Methods:**

FoxO1 agonists were identified by in silico screening and molecular dynamics simulation. Western blotting and reverse transcription‐quantitative polymerase chain reaction assays were used to assess protein and gene expression levels of P21, BIM, and PPARγ downstream of FoxO1 in SH‐SY5Y cells, respectively. Western blotting and enzyme‐linked immunoassays were performed to explore the effect of FoxO1 agonists on APP metabolism.

**Results:**

N‐(3‐methylisothiazol‐5‐yl)‐2‐(2‐oxobenzo[d]oxazol‐3(2H)‐yl) acetamide (compound D) had the highest affinity for FoxO1. Compound D activated FoxO1 and regulated the expression of its downstream target genes, *P21*, *BIM*, and *PPARγ*. In SH‐SY5Y cells treated with compound D, BACE1 expression levels were downregulated, and the levels of Aβ_1‐40_ and Aβ_1‐42_ were also reduced.

**Conclusions:**

We present a novel small‐molecule FoxO1 agonist with good anti‐AD effects. This study highlights a promising strategy for new drug discovery for AD.

## Graphical abstract

Through in silico and in vitro, N‐(3‐methylisothiazol‐5‐yl)‐2‐(2‐oxobenzo[d] oxazol‐3 (2H)‐yl) acetamide (compound D), as a novel FoxO1 agonist, can downregulate the levels of Aβ_1‐40_ and Aβ_1‐42_ by inhibiting BACE1. Compound D could be useful in the treatment of AD.

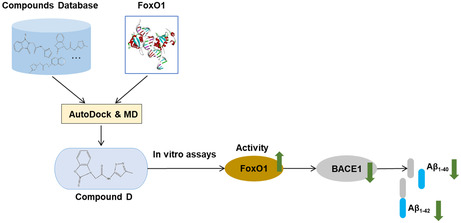

## 1 INTRODUCTION

Alzheimer's disease (AD) is a common neurodegenerative disease among elderly individuals. Approximately 50 million people globally have dementia, and 50–70% of these cases are attributed to AD.^1^ The World Health Organization reports that approximately 9.9 million new cases of dementia are diagnosed each year, and the total number of people with dementia is predicted to reach 75 million by 2030.^2^ Patients with AD suffer from behavioral decline and cognitive loss, such as memory and language impairment, and behavioral disturbances.^3^, ^4^ Therefore, the treatment of AD has been a hot research topic in the fields of drug discovery and clinical research over the last 20 years.

The anti‐AD drugs currently used in the clinical treatment are mainly cholinesterase inhibitors^5^ and *N*‐methyl‐d‐aspartate receptor antagonists.^6^ However, although these drugs alleviate the symptoms of AD, they cannot reverse the pathological process of the disease.^7^ Many studies have suggested that the deposition of β‐amyloid (Aβ) plaques is a key component in the development of AD.^8^ Aβ peptides, including Aβ_1‐40_ and Aβ_1‐42_, are produced through proteolytic processing of the amyloid precursor protein (APP) by β‐ and γ‐secretase. These peptides play an important role in AD.^9^ Therapeutic strategies targeting the amyloid cascade reduce Aβ levels; this is a potentially crucial step in the treatment of AD. β‐site APP cleavage enzyme 1 (BACE1) is the main β‐secretase, and downregulation of its activity could reduce Aβ production.^10^ Therefore, new lead drugs for AD that bind to new therapeutic targets are urgently required.

FoxO1, the most important transcription factor in the FoxO family, plays an important role in regulating cell proliferation, oxidative stress, cerebral ischemia, autophagy, and neurodegenerative diseases.^11^, ^12^, ^13^ FoxO1 is expressed in different brain regions, especially in the hippocampus.^14^ In our previous study, FoxO1 levels were found to be significantly lower in the cortical tissue of 6‐month‐old APP/presenilin 1 (PS1) transgenic mice compared to wild‐type mice. Overexpression of FoxO1 in an AD cell model effectively reduces the levels of Aβ and Tau phosphorylation.^15^ These results suggest that FoxO1 may be a potential therapeutic target for AD. However, there are no FoxO1 agonists available for the treatment of AD.

Virtual screening is one of the most effective drug discovery approaches. The advantages of molecular docking include fast and low‐cost high‐throughput screening, which can identify the binding model between a ligand and its targets. In this study, we employed molecular docking and molecular dynamics simulations to identify small molecules targeting FoxO1, thereby reducing the levels of Aβ through the downregulation of BACE1.

## 2 MATERIALS AND METHODS

### 2.1 Molecular docking

Molecular docking is a method used in drug discovery. It is based on the properties of the interaction mechanism between the receptor and its ligands.^16^ As a theoretical simulation method, it can predict binding modes and affinity. In recent years, molecular docking methods have become important techniques in computer‐aided drug discovery.^17^ In this study, molecular docking was performed using AutoDock Vina 1.1.2^18^ and PyMol version 2.4.0 software.^19^ Briefly, the three‐dimensional (3D) structure of FoxO1 (PDB code: 4lg0) was downloaded from the Research Collaboratory for Structural Bioinformatics Protein Data Bank (www.rcsb.org/pdb). The selection of the structures was based on the receptor model, resolution, and source organism. The structure 4lg0 had two chains determined by X‐ray diffraction at a resolution of 2.19 Ă. The protein was prepared by removing the ligands and bound water molecules in AutoDock Vina. The binding energies of the macromolecule coordinates and docking target site were evaluated using AutoGrid. In our previous study, we identified 11 compounds with potential activity against the FoxO family of transcription factors. The small molecules investigated in the current study included 11 compounds, namely, N‐((3‐methyl‐1,2,4‐oxadiazol‐5‐yl)methyl)‐3‐(thiophen‐2‐yl)‐1H‐pyrazole‐5‐carboxamide (compound A), 2‐(3‐oxo‐[1,2,4]triazolo[4,3‐a]pyrimidin‐2(3H)‐yl)‐N‐phenylacetamide (compound B), N‐((5‐methyl‐1,3,4‐oxadiazol‐2‐yl)methyl)‐5‐(thiophen‐2‐yl)‐1H‐pyrazole‐3‐carboxamide (compound C), N‐(3‐methylisothiazol‐5‐yl)‐2‐(2‐oxobenzo[d]oxazol‐3(2H)‐yl) acetamide (compound D), 2‐(1,3‐dioxoisoindolin‐2‐yl)‐N‐(thiazol‐2‐yl) acetamide (compound E), 3‐(pyrazin‐2‐yloxy)‐N‐(thiophen‐2‐yl) pyrrolidine‐1‐carboxamide (compound F), 2‐([1,2,4]triazolo[4,3‐a]pyridin‐3‐ylthio)‐N‐(5‐methylisoxazol‐3‐yl) acetamide (compound G), 2‐(4‐oxobenzo[d][1,2,3]triazin‐3(4H)‐yl)‐N‐(thiazol‐2‐yl) acetamide (compound H), N‐(pyridin‐2‐yl)‐2‐(5‐(thiophen‐2‐yl)‐isoxazol‐3‐yl) acetamide (compound I), N‐(5‐methyl‐1,3,4‐thiadiazol‐2‐yl)‐2‐(naphthalen‐2‐yloxy) acetamide (compound J), and N‐(2‐(4‐(3‐methyl‐1,2,4‐oxadiazol‐5‐yl)‐1H‐1,2,3‐triazol‐1‐yl) ethyl) benzamide (compound K). AutoDock Vina was used to calculate the binding free energy of the compounds in the macromolecular structure and predict the binding model between the receptor and ligands.

### 2.2 Molecular dynamics simulation validation by GROMACS

The results of virtual screening and molecular docking studies were validated by molecular dynamics simulation analysis that computationally simulates the motion of small molecules in biological environments. Molecular dynamics simulation was performed using GROMACS version 2019.6 software,^20^, ^21^ and the physical conditions were set to a constant temperature (305 K) and pressure (101 kPa). After the states of all environments were in equilibrium, compounds D, E, and J were bound to FoxO1 and observed every 10 ps (1 s = 1012 ps).^22^ We analyzed the simulation trajectories using the GROMACS analysis tools. In addition, the built‐in GROMACS program was used to analyze and visualize the molecular dynamics simulation results, as indicated by the root mean square deviation (RMSD), root mean square fluctuation (RMSF), and radius of gyration (Rg).

### 2.3 Preparation of compound D

We synthesized compound D, which showed the highest binding to FoxO1 The synthetic route of compound D was shown in Figure S1. Compound D was dissolved in dimethyl sulfoxide (DMSO) (Sigma‐Aldrich) to prepare a 10 mM drug stock. Nuclear magnetic resonance (NMR) and mass spectrometry (MS) analyses showed that the analytical and spectral data were in agreement with the assigned structure of compound D (Figures [Link], [Link]).

### 2.4 Cell culture

SH‐SY5Y cells were kindly provided by Professor Dehua Chui of the Neuroscience Research Institute, Peking University. The cells were cultured in Dulbecco's modified Eagle medium (DMEM)/F12 medium (Gibco) supplemented with 10% (v/v) heat‐inactivated fetal bovine serum (FBS) (Gibco) and a penicillin–streptomycin mixture (1:100; Gibco) in an incubator at 37°C in a humidified atmosphere of 95% air and 5% CO_2_.

### 2.5 Cell morphology and cell viability assays

Cultured cells were inoculated at a density of 1 × 10^6^ cells per well in six‐well plates containing DMEM. After 24 h of incubation, the medium was replaced with fresh medium. Cells were subsequently treated with different concentrations of compound D (0, 20, 40, 60, 80, and 100 μM) for 24 h. After incubation, cell morphology was observed and photographed using an inverted microscope (Ts2R‐FL, Nikon).

To explore the effects of different concentrations of compound D on cell viability, a cell‐counting kit‐8 (CCK‐8; #HY‐K0301; MedChemExpress) assay was performed to assess cell proliferation and toxicity. Following the manufacturer's instructions, SH‐SY5Y cells were pre‐incubated in 96‐well plates at 37°C for 24 h and then treated with different doses of compound D (0, 20, 40, 60, 80, and 100 μM) for 24 h. Then, 10 μL of CCK‐8 reagent was added to each well, and the cells were incubated for 4 h. The absorbance of the samples was measured at 450 nm on a microplate reader.

### 2.6 Reverse transcription‐quantitative polymerase chain reaction

The expression of related genes in SH‐SY5Y cells was detected using reverse transcription‐quantitative polymerase chain reaction (RT‐qPCR). SH‐SY5Y cells were treated with different concentrations (0, 20, 40, 60, 80, and 100 μM) of compound D for 24 h, and total RNA was isolated using RNAiso Plus (# 9108; Takara Biotechnology, Co., Ltd.) according to the manufacturer's instructions. Immediately after extraction, the RNA concentration of each sample was measured, and an equal amount of total RNA (1 μg) was reverse‐transcribed to cDNA using the PrimeScript RT Reagent kit with gDNA Eraser (# RR047A; Takara Biotechnology Co., Ltd.) according to the manufacturer's instructions. RT‐qPCR was performed in a 20 μL volume with 2 μL of cDNA, 0.4 μM sense and antisense primers, and 10 μL of TB Green Premix Ex Taq II (# RR820A; Takara Biotechnology Co., Ltd.) on a QuantStudio™ 5 Real‐Time PCR Instrument (Applied Biosystems; Thermo Fisher Scientific, Inc.). The reactions were performed according to the manufacturer's protocol, using a three‐step PCR amplification method. The first step was predenaturation at 95°C for 30 s; the second step involved 40 cycles of denaturation at 95°C for 5 s and annealing at *T*_m_ °C for 34 s; and the third step involved 15 cycles of 95°C for 30 s, 60°C for 1 min, and 95°C for 15 s. The details of the primer sequences are presented in Table 1. The average gene expression level after β‐actin normalization was used as a calibrator to determine the relative levels of the target genes. Each sample was analyzed in triplicate. The relative expression levels of the target genes were calculated using the 2^−ΔΔCt^ method.^23^

**TABLE 1:** Primer sequences used in this study.

Gene name	Primer (5′ to 3′)
Human	
*P21*	Forward: GGATGTCCGTCAGAACCCA
	Reverse: CCTGCCTCCTCCCAACTCA
*BIM*	Forward: TCATCGCGGTATTCGGTTCG
	Reverse: CCTGCCTCATGGAAGCTTGT
*PPARγ*	Forward: GTGGACATCCGCAAAGACC
	Reverse: CGGACTCGTCATACTCCTGCT
*β‐Actin*	Forward: GTGGACATCCGCAAAGACC
	Forward: CGGACTCGTCATACTCCTGCT

### 2.7 Western blotting

SH‐SY5Y cells were cultured in six‐well plates with 1 × 10^6^ cells per well and then treated with different concentrations of compound D (0, 20, 40, 60, 80, and 100 μM). After 24 h, the cells were lysed with radioimmunoprecipitation assay buffer and subjected to 10% sodium dodecyl sulfate‐polyacrylamide gel electrophoresis for western blotting. The proteins were transferred onto a methanol‐charged polyvinylidene fluoride (PVDF) membrane and probed with anti‐FoxO1 (1:1000, # 2880 S; Cell Signaling Technology), anti‐P21 (1:1000, # 2947 S; Cell Signaling Technology), anti‐BIM (1:1000, # 22037‐1‐AP; Proteintech), anti‐PPARγ (1:1000, # sc‐7273; Santa Cruz Biotechnology), anti‐BACE1 (1:1000, # 5606 S; Cell Signaling Technology), anti‐APP (1:10,000, # A8717; Sigma–Aldrich), anti‐ADAM10 (1:1000, # 14194 S; Cell Signaling Technologies), anti‐PS1 (1:1000, # ab176560; Abcam), anti‐p‐FoxO1 (1:1000, # 9461 S; Cell Signaling Technology), or anti‐GAPDH (1:5000, # AB0037; Abways Technology) antibodies. After incubation with the primary antibody, the membranes were washed with Tris‐buffered saline with Tween 20 (1× TBST) thrice for 10 min each and then incubated with the appropriate secondary antibody for 1.5 h at 37°C on a shaker. The membranes were washed again with TBST (1×) four times prior to imaging with enhanced chemiluminescence (# WBKLS100, Immobilon Western HRP Substrate, Milipore). The blot images were acquired using gel documentation system （chemi XX9, Syngene). The densities of protein bands were analysed by an image analysis software (Image J, National Institutes of Health).

### 2.8 Measurement of Aβ levels using enzyme‐linked immunosorbent assay

After 24 h of treating SH‐SY5Y cells with compound D in the range of 0–100 μM, the medium was harvested and analyzed. The levels of human Aβ_1‐40_ and Aβ_1‐42_ in the cells, and the medium were determined using sandwich enzyme‐linked immunosorbent assay (ELISA) kits (#E‐EL‐H0542c, #E‐EL‐H0543c, Elabscience) according to the manufacturer's instructions. The data were normalized to the data from the control cells. All ELISAs were performed in triplicate.

### 2.9 Statistical analyses

Data are presented as mean ± standard error of mean (SEM) and were tested for normality before statistical analysis. Differences between experimental groups were determined by one‐way analysis of variance, and Tukey's post hoc test was used for multiple comparisons. All results were analyzed using GraphPad Prism software (PC version 7.0; GraphPad) and considered statistically significant at *p* < 0.05.

## 3 RESULTS

### 3.1 Results of virtual screening

**FIGURE 1:**
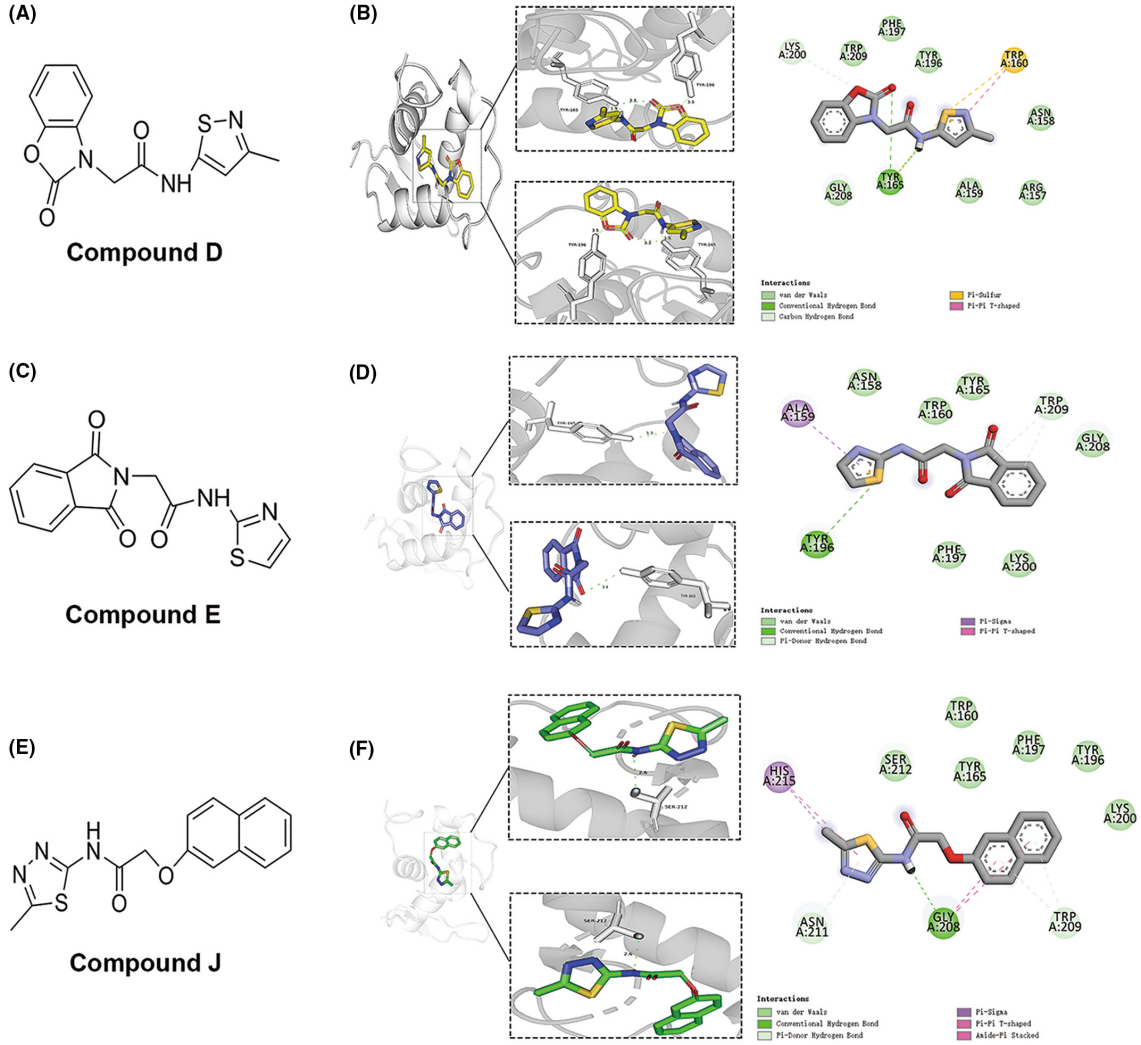
Computational docking of compounds D, E, and J to FoxO1. (A, C, E) Comparison of the molecular structures of compounds D, E, and J. (B, D, F) The three‐ and two‐dimensional molecule interaction maps were generated based on the calculated binding between FoxO1 and compounds D, E, and J. The green line represents the presumed hydrogen bond, light green represents van der Waals forces, purple represents pi–sigma interaction, and light pink represents pi‐alkyl and pi–pi bonds.

Free energy was calculated using AutoDock Vina (Table 2). The free energies of compounds D, E, and J were higher than those of the other compounds. The docking models of compounds D, E, and J with FoxO1 are shown in Figure 1. The binding energies were −7.4 kcal/mol, −6.9 kcal/mol, and −7.1 kcal/mol, respectively. Compounds D, E, and J have an acetamide basic structure; however, compound D differs from the other two compounds as it also contains a benzoxazole structure (Figure 1). Compound D interacted with the amino acid residue Tyr‐165 of the A chain in the target protein to form two conventional hydrogen bonds (Figure 1A,B). The S atom of compound E formed a conventional hydrogen bond and a pi–σ bond with amino acid residues Tyr‐196 and Ala‐159 of the A chain in the target protein, respectively (Figure 1C,D). The oxygen atom of compound J formed one conventional hydrogen bond with Gly‐208 of the A chain in the target protein (Figure 1E,F).

**TABLE 2:** Results of virtual screening.

Compounds	CAS number	MW	FoxO1 docking energy (kcal/mol)
a	1297612‐99‐7	289.31	−6.8
b	1327592‐50‐6	299.35	−6.5
c	1240264‐37‐2	289.31	−6.7
d	1207042‐01‐0	289.31	−7.4
e	65919‐36‐0	287.29	−6.9
f	2034280‐85‐6	290.34	−6.2
g	670269‐98‐4	289.31	−6.3
h	904011‐80‐9	287.30	−6.9
i	953183‐77‐2	285.32	−6.6
j	448914‐23‐6	299.35	−7.1
k	2034508‐82‐0	298.30	−6.9

Abbreviations: CAS, chemical abstracts service; MW, molecular weight.

### 3.2 Molecular dynamics simulations of FoxO1–compound D

**FIGURE 2:**
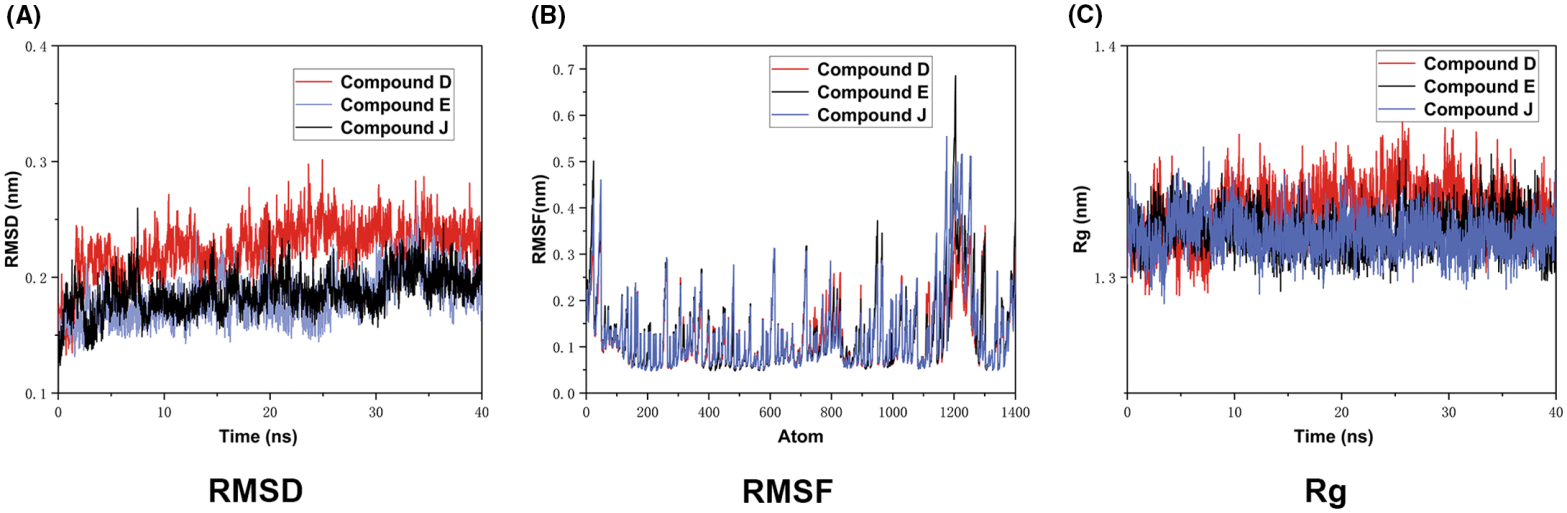
The results of molecular dynamics simulation of compound D–FoxO1 complexes. Comparison of (A) the root mean square deviation (RMSD) of backbone atoms, (B) the root mean square fluctuation (RMSF), and (C) the radius of gyration (Rg) potential energy values for 40 ns molecular dynamics simulations for three systems.

The RMSD curves of the compound D–FoxO1 complex were initially stable, and structural integrity was maintained for most of the simulation time. Compounds E and J also maintained structural integrity for most of the simulation time. However, a significant increase in the RMSD values was observed for compounds E and J at 20 ns. Although the levels of RMSD fluctuation were not high, the remaining simulation traces for both compounds exhibited low RMSD values between 0 and 25 nm, which may account for their lower flexibility than compound D throughout the simulation time (Figure 2A). Although the compound D–FoxO1 complex showed initial fluctuations in the RMSD plot, it remained stable throughout the simulation for 40 ns (Figure 2A). The RMSF values of the Cα atoms of the three docking complexes were subsequently calculated, as displayed in Figure 2B. We observed large fluctuations at the extreme ends, which is a typical phenomenon. The RMSF values rarely crossed 0.3 nm for most of the atoms. We noted high residual fluctuations in the N‐terminal region of FoxO1 in the presence of compounds E and J and in the C‐terminus of FoxO1 in the presence of compound D. As shown in the RMSF plot, amino acids located at residues 1000–1200 (within the catalytic domain) showed more deviations, reflecting the flexibility of this region of the FoxO1 protein. The Rg enables the assessment of changes in the compactness of protein–ligand complexes. The average packing density of each protein complex decreased in the complexed state (Figure 2C). The graph presented in Figure 2 shows that the stability of FoxO1 complexed with compound D was slightly higher than the stability of FoxO1 complexed with compound E or J. Thus, the trajectory results suggest that compound D reliably targets FoxO1. Additionally, the stability of the systems strengthened the credibility of the docking results.

### 3.3 Effect of compound D on SH‐SY5Y cell viability

**FIGURE 3:**
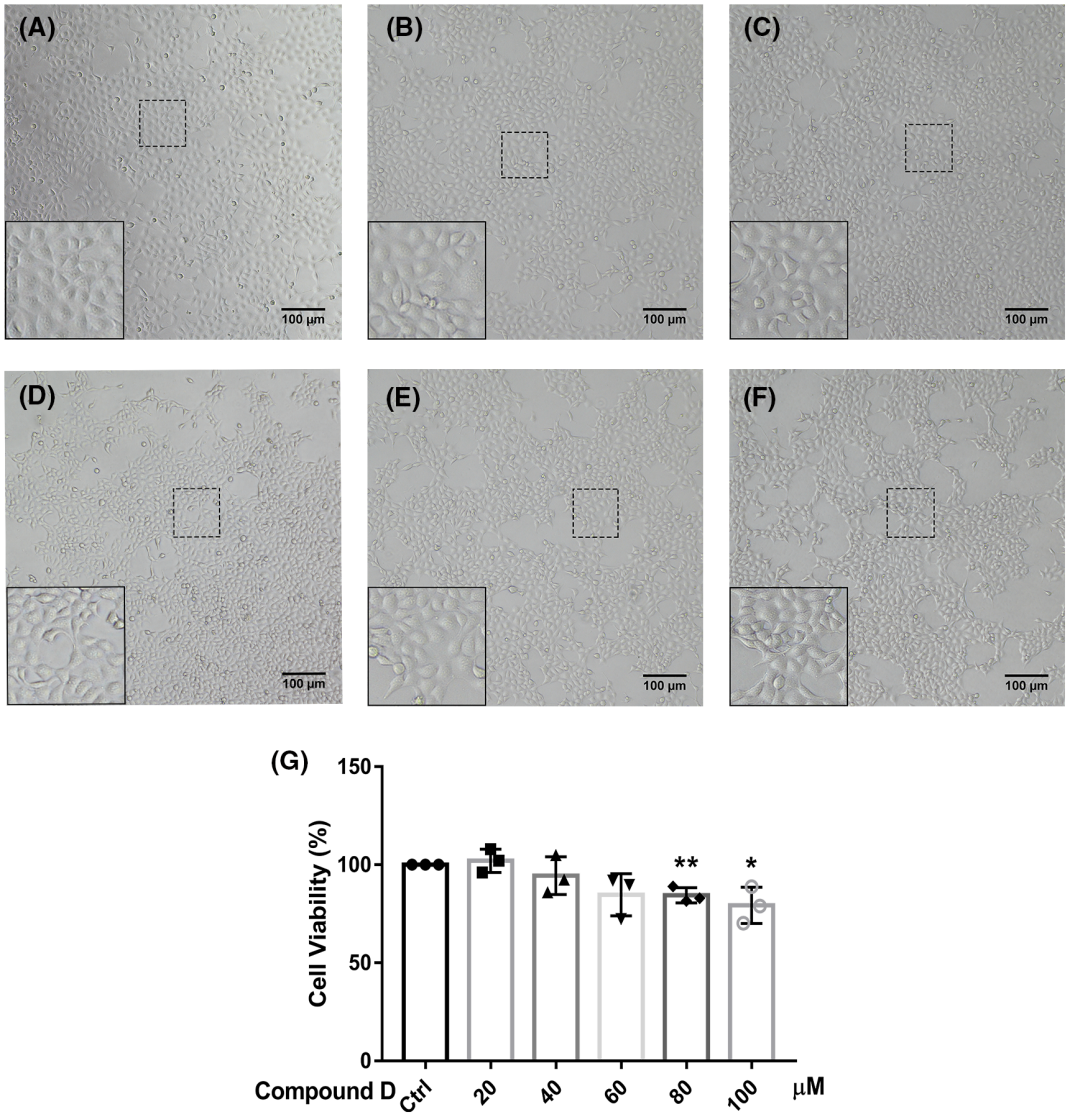
Compound D affects the viability of SH‐SY5Y cells. (A–F) SH‐SY5Y cells were treated with the compound D (A, 0 μM; B; 20 μM; C, 40 μM; D, 60 μM; E, 80 μM; F, 100 μM). The scale bar represents 100 μm. (G) The viability of SH‐SY5Y cells was determined using the cell‐counting kit‐8 assay. The data are shown as the mean ±  SEM of three independent experiments. *p < 0.05, **p < 0.01, ***p < 0.001 versus control cells.

Cell density after treatment with compound D was also determined by observation under a microscope. The results were consistent with the results of the CCK‐8 assay. As shown in Figure 3A–F, the cells showed obvious apoptotic symptoms, such as nuclear fixation and fragmentation, 24 h after treatment with 80 or 100 μM compound D. The number of SH‐SY5Y cells decreased sequentially after treatment with 20, 40, 60, 80, and 100 μM for 24 h, indicating that the effects of compound D on SH‐SY5Y cell activity were concentration‐dependent. The CCK‐8 assay was used to detect changes in the viability of SH‐SY5Y cells treated with different concentrations of compound D. As shown in Figure 3G, the viability of SH‐SY5Y cells treated with compound D decreased gradually with increasing concentrations compared with the viability of control cells.

### 3.4 Compound D enhances the activity of FoxO1

**FIGURE 4:**
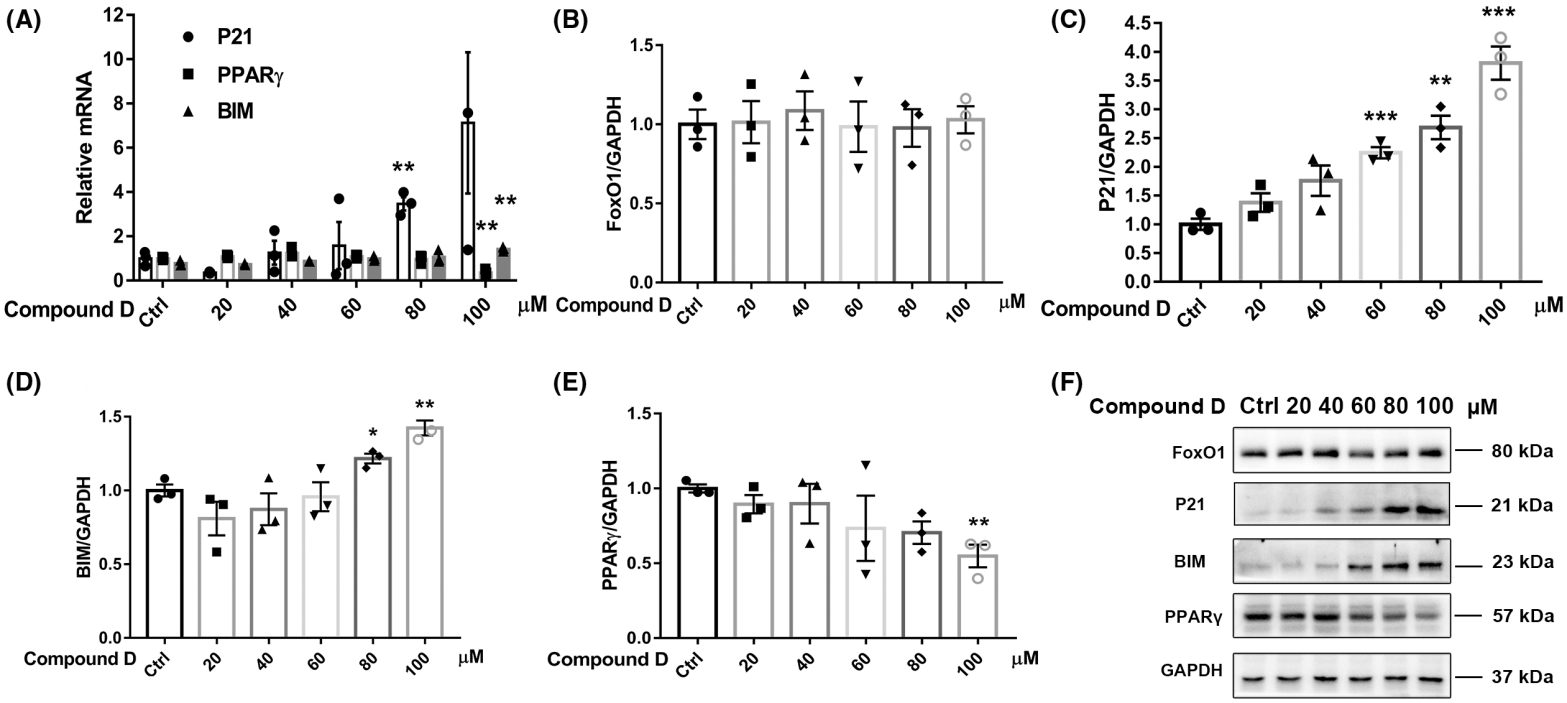
Compound D enhances the transcriptional activity of FoxO1. (A) Reverse transcription‐quantitative polymerase chain reaction analysis of P21, BIM, and PPARγ gene expression. (B–F) Western blotting analysis of FoxO1, P21, BIM, and PPARγ expression in SH‐SY5Y cells after treatment with different concentrations of compound D. The data are shown as the mean ± SEM of three independent experiments. *p < 0.05, **p < 0.01, ***p < 0.001 versus control cells.

**FIGURE 5:**
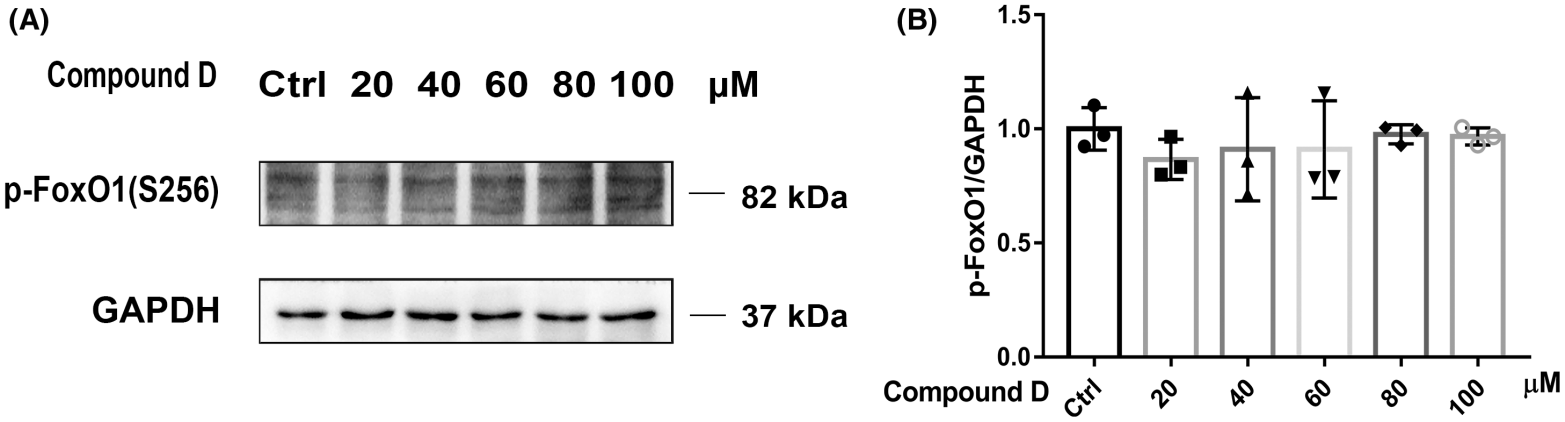
Effect of compound D on the levels of p‐FoxO1 in SH‐SY5Y cells. (A, B) Western blotting analysis of p‐FoxO1 levels in SH‐SY5Y cells after treatment with different concentrations of compound D. The data are shown as the mean ± SEM of three independent experiments. *p < 0.05, **p < 0.01, ***p < 0.001 versus control cells.

The effect of compound D on the activity of FoxO1 was investigated further. RT‐qPCR and western blotting analyses showed that compound D did not increase FoxO1 protein expression levels (Figure 4B,F) and did not significantly change p‐FoxO1 protein levels (Figure 5A,B) but significantly increased the protein and transcript levels of genes downstream of FoxO1, namely, *P21*, *BIM*, and *PPARγ*, in a concentration‐dependent manner (Figure 4A). As shown in Figure 4A, *P21* and *BIM* transcript levels gradually increased, and *PPARγ* transcript levels gradually decreased with increasing concentrations of compound D. Western blotting results (Figure 4C–F) showed that changes in protein expression levels were consistent with the changes in transcript levels. These results indicate that compound D is a FoxO1 agonist that significantly enhances *FoxO1* activity.

### 3.5 Compound D reduces Aβ production by inhibiting the expression of BACE1

**FIGURE 6:**
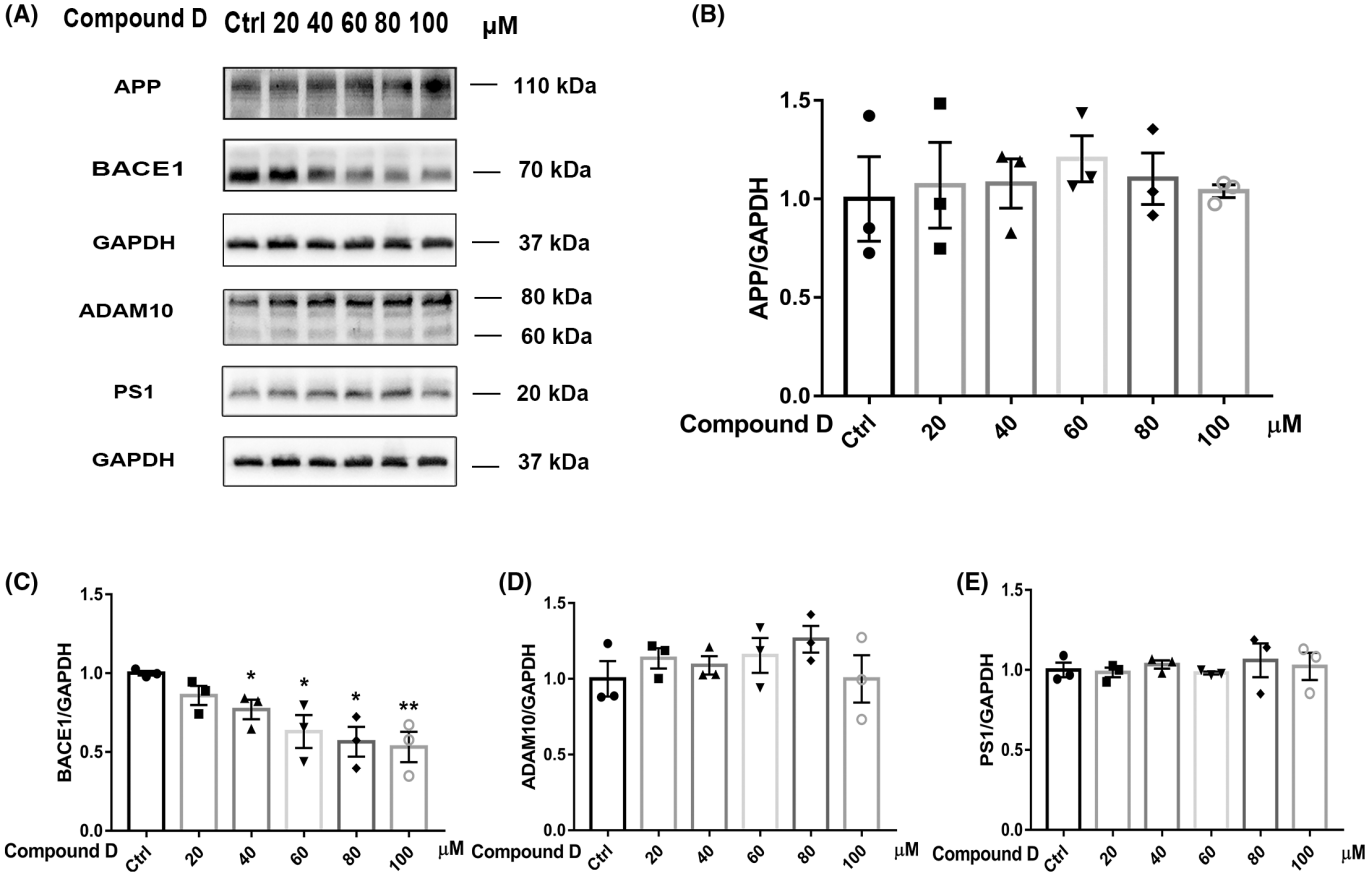
Effect of compound D on the levels of APP metabolic pathway components in SH‐SY5Y cells. (A–E) Western blotting analysis of APP, BACE1, ADAM10, and PS1 expression in SH‐SY5Y cells after treatment with different concentrations of compound D. The data are shown as the mean ± SEM of three independent experiments. *p < 0.05, ** represents *p <* 0.01, ***p < 0.001 versus control cells.

**FIGURE 7:**
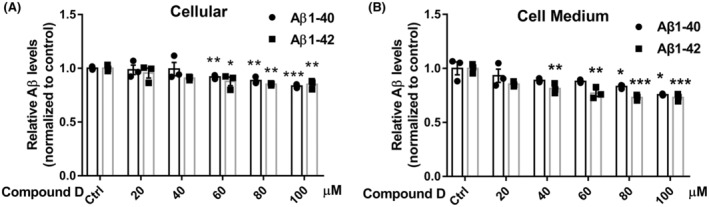
Effect of compound D on Aβ levels in SH‐SY5Y cells. (A) Cellular Aβ_1‐40_ and Aβ_1‐42_ levels. (B) Aβ_1‐40_ and Aβ_1‐42_ levels in the cell culture medium. The data are shown as the mean ± SEM of three independent experiments. *p < 0.05, **p < 0.01, ***p < 0.001 versus control cells.

In a previous experiment, we showed that compound D activates FoxO1 activity. To further investigate the mechanism of action of compound D, we examined the levels of the Aβ‐processing‐related proteins, ADAM10, PS1, APP, and BACE1 (Figure 6A). The expression level of BACE1 decreased significantly with increasing compound D concentrations (Figure 6A,C). However, compound D did not affect the expression of APP, ADAM10, and PS1 (Figure 6B,D,E). We then assessed whether compound D treatment reduced Aβ formation in SH‐SY5Y cells. We found that the levels of Aβ_1‐40_ and Aβ_1‐42_ in cell supernatants and cellular significantly decreased with increasing concentrations of compound D (Figure 7A,B). Overall, compound D downregulated the expression of BACE1 in SH‐SY5Y cells, thereby downregulating the intracellular and extracellular levels of Aβ_1‐40_ and Aβ_1‐42_. Taken together, these results indicate that as a FoxO1 agonist, compound D is a potential drug for the treatment of AD.

## 4 DISCUSSION

In the present study, a novel FoxO1 agonist, compound D, was identified through in silico screening coupled with bio‐evaluation. Compound D demonstrated anti‐AD effects by inhibiting the Aβ enzymatic pathway involving BACE1, thereby reducing Aβ levels. FoxO1 activity can be modulated by the PI3K/Akt pathway in many cell and mouse models.^24^, ^25^ Moreover, FoxO1 activity can also be regulated by P38 and Erk.^26^ However, effective FoxO1 agonists remain unknown. Some small molecules indirectly affect the expression of FoxO1 by activating the signaling pathway upstream of FoxO1, thereby regulating FoxO1 activity.^27^, ^28^, ^29^ For example, resveratrol protects rats against severe acute pancreatitis by activating the SIRT1–FOXO1 axis.^27^ We previously demonstrated that FoxO1 activity played an important role in APP processing related to AD. Thus, FoxO1 may be a potential target for the treatment of AD. Using a combination of computer‐aided drug design and biological experiments, we discovered a small molecule that directly activates FoxO1.

In recent years, an increasing number of drugs have been screened using computer‐aided drug design technology, which can effectively screen small‐molecule drugs that bind to their target, achieving rapid selection of leading drug compounds.^30^, ^31^ Virtual screening results were validated using molecular dynamics simulations to ensure their reliability and confirm the molecular interactions.^30^ We aimed to discover lead compounds targeting FoxO1 more efficiently through virtual screening, followed by molecular dynamics simulations. The combination of these methods can greatly improve the efficiency of drug discovery. In silico screening, experiments showed that compound D bound to FoxO1 at Tyr‐165. In addition, other compounds may bind to FoxO1 at Tyr‐196 and Ala‐159. Tyr‐165 is the DNA‐binding site of FoxO1, and Tyr‐196 and Ala‐159 are in the Fork‐head domain as shown in the uniprot.^32^ In silico docking studies have shown that ferulic acid, exhibiting anti‐inflammatory, antidiabetic, cardioprotective, and neuroprotective activities, also interacts with FoxO1 with maximum binding affinity at Tyr‐165.^33^, ^34^ Therefore, we speculated that Tyr‐165 might be an important FoxO1 agonist binding site. These findings may facilitate the further development of novel FoxO1 agonists.

When simulating the stable state of the docked complex in the native environment, the compound D–FoxO1 complex exhibited excellent molecular dynamics simulation results. To promote the most stable binding conformation, a simulation time of 40 ns was used in this study to allow side chain reorganization.^35^, ^36^ In addition, a phospholipid bilayer environment was chosen, as in previous studies.^22^, ^37^ Throughout the simulation, compound D remained relatively stable compared with the other compounds. Moreover, compound D had the highest binding free energy with FoxO1 (−7.4 kcal/mol) and was bound more tightly than the other two ligands.

As a transcription factor, FoxO1 regulates the expression of downstream genes. There is no specific FoxO1 activity assay kit available; therefore, we investigated FoxO1 activity by testing the expression of FoxO1 target genes. Several transcription factor studies have demonstrated MHY2013 as a potent pan‐agonist of PPAR, significantly elevating the mRNA levels of PPARα target genes (*ACOX1* and *CPT1*).^38^ In our study, P21 and BIM, downstream targets of FoxO1, were activated by FoxO1 upregulation.^39^, ^40^ Alternatively, the expression level of the downstream target, PPARγ,^41^, ^42^ gradually decreased in response to compound D binding. FoxO1 directly activates *BIM* and *P21* gene expression.^40^, ^43^ FoxO1 is primarily localized in the nucleus where it mediates normal physiological functions. When FoxO1 is phosphorylated and translocates from the nucleus to the cytoplasm, its transcriptional activity is downregulated.^44^ FoxO1 or p‐FoxO1 levels did not change significantly with increasing concentrations of compound D, whereas the expression levels of BIM and P21 significantly increased, and the level of PPARγ gradually decreased. These results suggest that FoxO1 is activated, with significantly higher transcriptional activity, in response to interference by compound D in a dose‐dependent manner. Our study may provide a new approach to the validation of transcription factor activity, which is of great interest to the field of targeted drug research.

We found that compound D significantly downregulated BACE1 activity, affecting Aβ metabolism; hence, compound D is essential for AD treatment. The pathogenesis of amyloid deposition begins with altered cleavage of APP at the plasma membrane by BACE1 and γ‐secretase, producing insoluble Aβ fibrils. The downregulation of BACE1 activity inhibits the cleavage of APP into Aβ. Many previous reports have indicated that BACE1 is a target for the treatment of AD. Several small molecules targeting BACE1 have been tested in the clinical trials for the treatment of AD.^45^, ^46^, ^47^ In this study, we found that compound D reduced BACE1 expression levels in a dose‐dependent manner, thereby downregulating Aβ levels. However, compound D had no significant effect on the expression of PS1, a member of the γ‐secretase family of AD‐related genes.^48^ ADAM10, a member of the constitutive α‐secretase and metalloproteinase ADAM family, plays a key role in reducing Aβ production. We also did not detect any significant difference in ADAM10 and APP expression levels after treatment with compound D. In our previous study, FoxO1 overexpression was found to downregulate BACE1, PS1, and APP expression levels.^15^ Therefore, we hypothesized that an FoxO1 agonist may affect FoxO1 activity in another way. For example, the FoxO1 agonist did not affect the location of FoxO1 or the level of FoxO1 in the cytoplasm and nucleus.

The results presented herein showed that the binding of compound D to the transcription factor FoxO1 may cause structural changes, thereby enhancing the in vitro activity of FoxO1 without changing the catalytic site. However, further research is needed to optimize the dose of compound D. Additionally, the structure of compound D may be modified to optimize its binding affinity and improve its efficacy. We aim to expand our screening to additional small molecule databases, discover more lead compounds, further validate their activity in vitro and in vivo, and explore specific mechanisms for the treatment of AD in animal models. More details about the mechanism of action of compound D will be investigated in our future work.

In this study, computational docking was performed using the entire FoxO1 structure. We discovered a new FoxO1 agonist in the compound library and validated it using in vitro biological experiments. This workflow combining virtual screening and in vitro experiments may provide a new reference for drug discovery. Compound D should be further investigated as a potential lead compound for the treatment of AD.

## 5 CONCLUSIONS

In this study, a novel FoxO1 agonist was identified through virtual screening and pharmacological experiments, providing new research methods and strategies for target drug studies. Compound D is the first specific FoxO1 agonist reported to downregulate BACE1 expression, leading to reduced Aβ levels and anti‐AD effects. Therefore, compound D may be a lead compound for the treatment of AD. The discovery of new drugs is essential for the improved treatment of AD.

## AUTHOR CONTRIBUTIONS

WZ, ZJY, and HCW conceived and designed the experiments; MTL, XWM, YTS, and YMZ performed and analyzed the data; TJN and YCD performed some of the experiments; MTL and HCW wrote the manuscript and prepared the figures; WZ, ZJY, MTL, and HCW revised the paper; and RLS and LLS interpreted the data. All authors reviewed and approved the final version of the article.

## CONFLICT OF INTEREST STATEMENT

All authors declare that they have no competing interests.

## Supporting information


Figure S1.

Figure S2.

Figure S3.

Appendix S1.

## Data Availability

The data originating from this study are included in the article and supplementary material. Further inquiries can be directed to the corresponding authors.
