# Supplementary figures and images for: In silico and in vitro analyses of a novel FoxO1 agonist reducing Aβ levels via downregulation of BACE1
Source: CNS Neurosci Ther. 2023 Mar 9;30(3):e14140. doi: 10.1111/cns.14140 (PMC10915984; doi:10.1111/cns.14140)

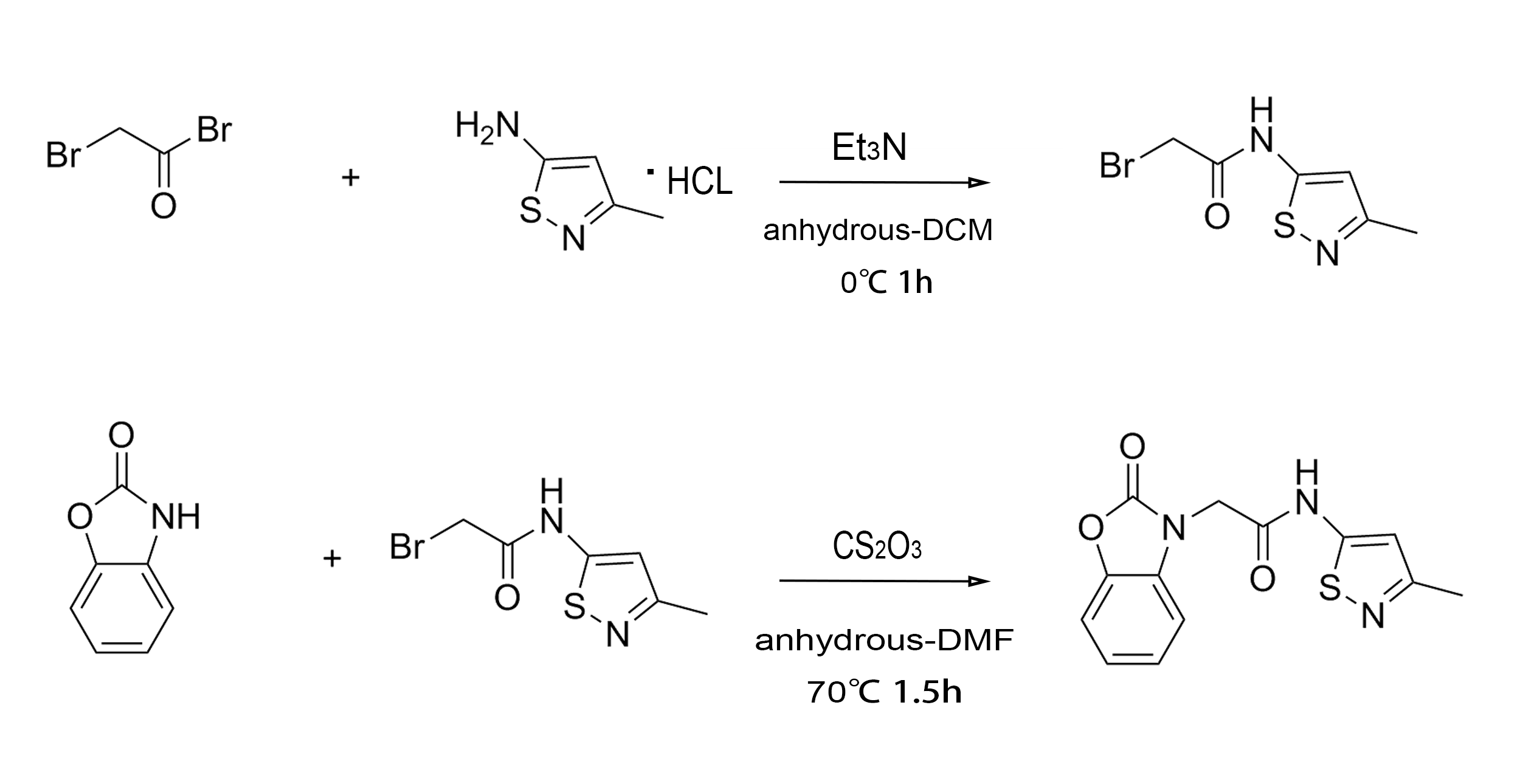

Supplement: Supplementary file 1 — Figure S1. [file CNS-30-e14140-s001.tif]

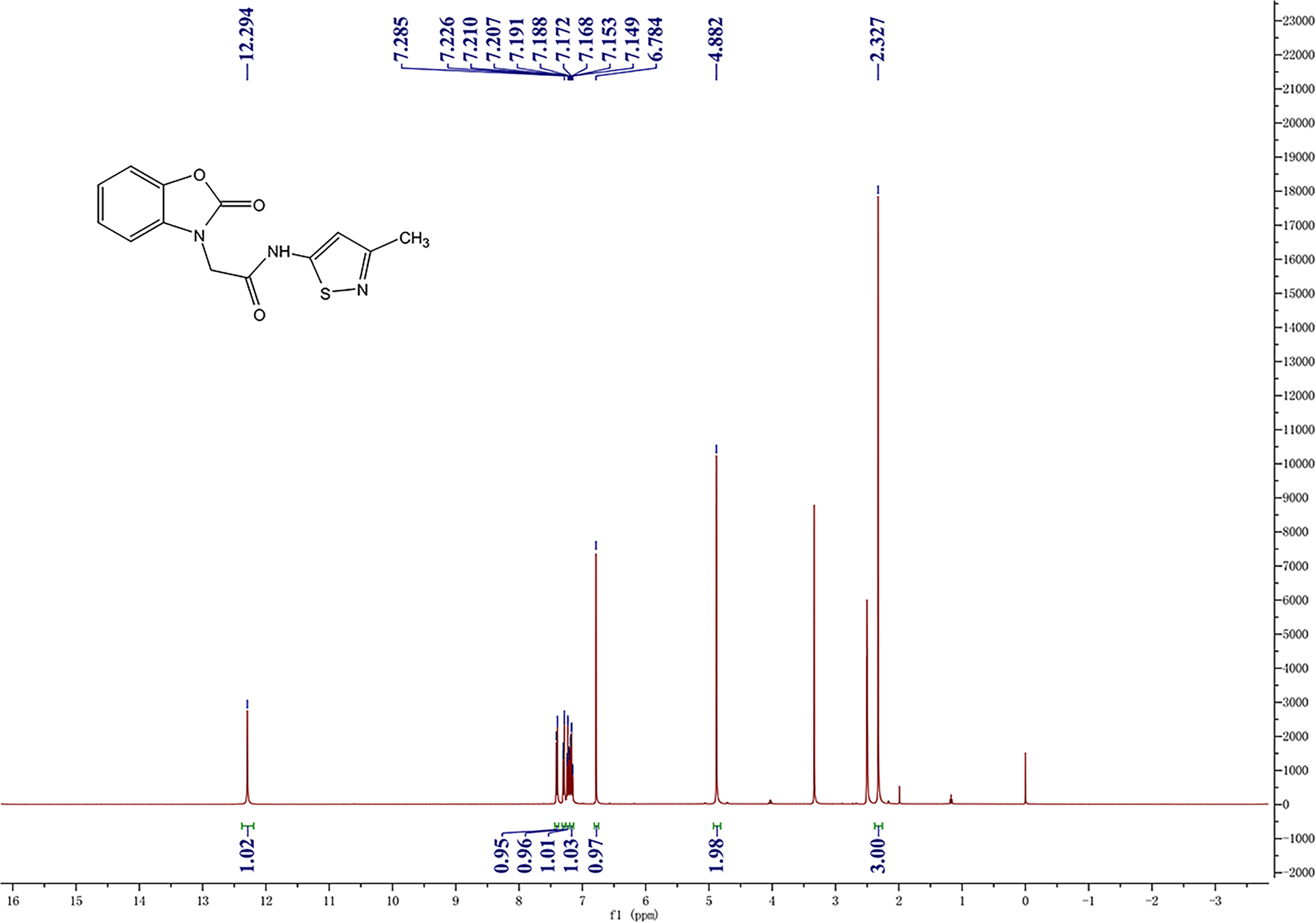

Supplement: Supplementary file 2 — Figure S2. [file CNS-30-e14140-s002.tif]

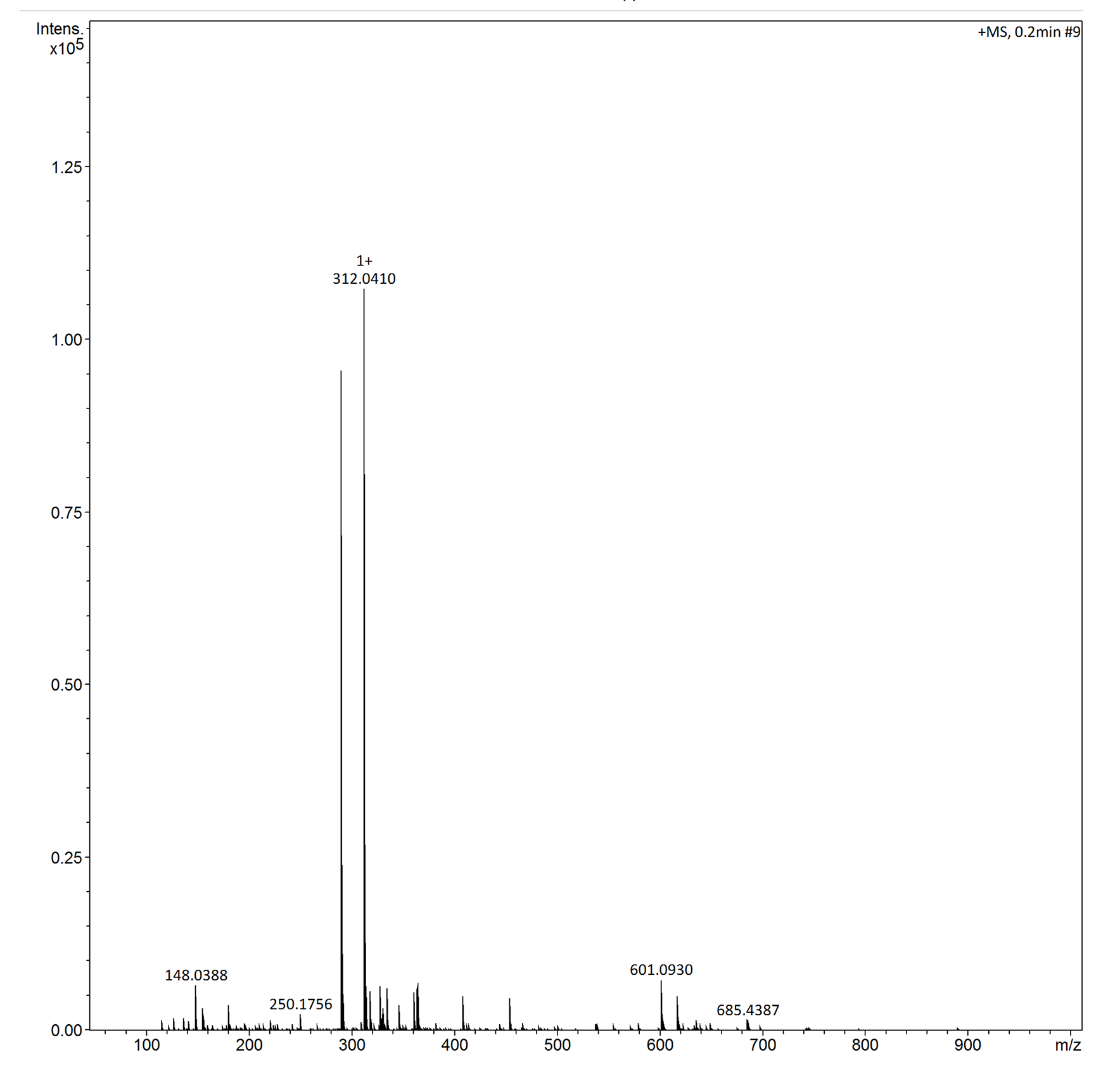

Supplement: Supplementary file 3 — Figure S3. [file CNS-30-e14140-s003.tif]
