# Supplementary material for: In silico and in vitro analyses of a novel FoxO1 agonist reducing Aβ levels via downregulation of BACE1
Source: CNS Neurosci Ther. 2023 Mar 9;30(3):e14140. doi: 10.1111/cns.14140 (PMC10915984; doi:10.1111/cns.14140)
Supplement: Supplementary file 4 — Appendix S1. [file CNS-30-e14140-s004.docx]

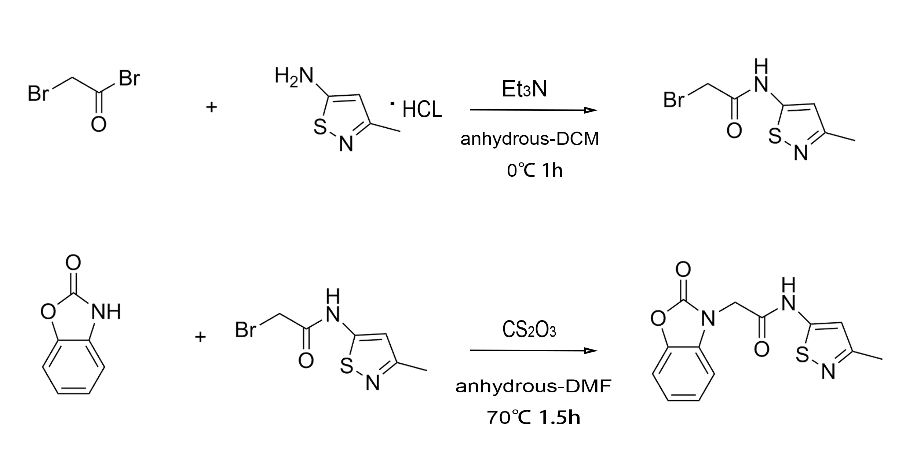


**Supplementary Figure S1.** The synthetic route of compound D


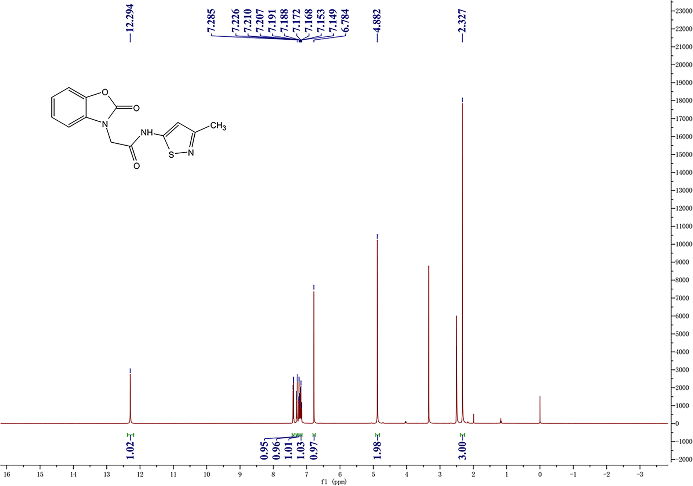


**Supplementary Figure S2.** Nuclear magnetic resonance analysis of compound D


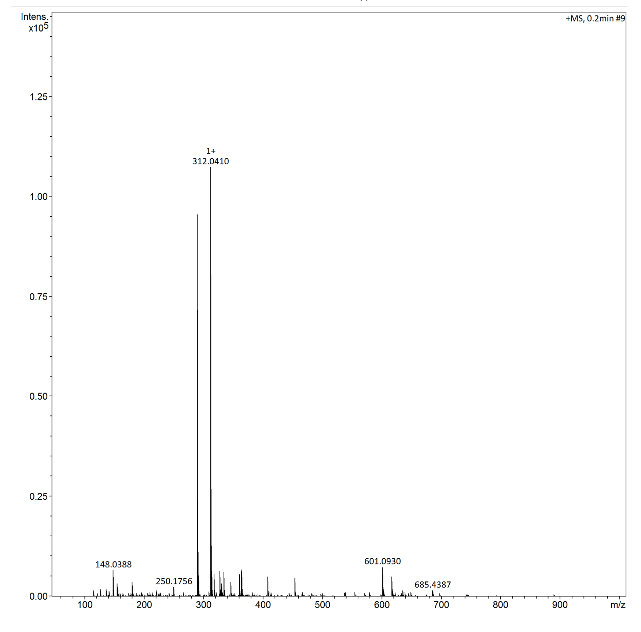


**Supplementary Figure S3.** Mass spectrometric analysis of compound D
